# Magnetophoretic Equilibrium of a Polydisperse Ferrofluid

**DOI:** 10.3390/nano11112849

**Published:** 2021-10-26

**Authors:** Andrey A. Kuznetsov, Ivan A. Podlesnykh

**Affiliations:** 1 Institute of Natural Sciences and Mathematics, Ural Federal University, 620000 Ekaterinburg, Russia; 2 Electrical Engineering Faculty, Perm National Research Polytechnic University, 614990 Perm, Russia; podlesnihwork@gmail.com

**Keywords:** magnetic nanoparticles, ferrofluid, magnetophoresis, diffusion, mass transfer, polydispersity

## Abstract

The equilibrium concentration distribution of magnetic nanoparticles in a nonuniform magnetic field is studied theoretically. A linear current-carrying wire is used as a source of a nonuniform field. An exact solution for the concentration profile of a dilute monodisperse suspension is obtained within the framework of the continuous mass transfer theory. The applicability of this solution in a broad range of amperage values is tested using Langevin dynamics simulations. Obtained solution is also generalized for polydisperse suspensions. It is demonstrated that the particle size distribution in a polydisperse system strongly depends on the distance from the wire and in general does not coincide with the original distribution of a uniform suspension.

## 1. Introduction

Modern day magnetic nanomaterials stay firmly at the forefront of innovation in biotechnology and medicine [1,2,3,4]. The physical basis for many of their perspective biomedical applications is the phenomenon of magnetophoresis, that is, the motion of magnetic objects under the action of a nonuniform magnetic field. For example, magnetic bioseparation—a medical diagnostics method, in which magnetic particles are first mixed with biological material obtained from the patient’s body; then the particles (due to the functionalized surface) attach to cells or biomolecules of a certain type (disease markers); and at the last stage, particles (together with biomarkers) are separated from the mixture using a gradient field [5]. A similar approach can be used to test the food quality [6]. For separation, both micro- and nanoparticles can be used, but the latter are preferable, since they have a higher binding capacity due to higher surface-to-volume ratio [7]. Particle capture can occur either from the static volume of the mixture [8] or from the flow [9]. Another technique based on magnetophoresis is the magnetic drug targeting [10]. The idea is to accumulate drug-loaded nanoparticles injected into the bloodstream near a pathological site (for example, a tumor) using a magnetic field gradient.

On the other hand, for classical designs of ferrofluid devices the redistribution of particles in a nonuniform field is highly undesirable. Ferrofluids (FFs) are colloidal solutions of single-domain magnetic nanoparticles (MNPs) in a nonmagnetic carrier fluid. MNPs are typically covered by a protective surfactant shell that protects them from irreversible aggregation (instead, electrostatic stabilization can also be used [11]). Although the particles do not precipitate due to the intense thermal motion, the presence of field gradients in the working volume of the FF device inevitably causes the dispersed phase to drift. In the absence of convective flows, the only process that prevents such a drift is the gradient Brownian diffusion. The competition between these two mechanisms leads to the slow establishment of a nonuniform concentration distribution of nanoparticles in the FF [12]. This phenomenon is similar to the establishment of a barometric distribution in a gravitational field. If the magnetic field gradient is large enough, then the degree of concentration inhomogeneity turns out to be significant, which is extremely undesirable from the applied point of view. Redistribution of particles critically affects the stability of the performance characteristics of FF seals and sensors [13,14].

Thus, magnetophoresis typically plays a significant role in the applications of MNPs. It is also known that mass transfer processes and equilibrium concentration distribution in FFs can be strongly affected by the particle polydispersity [15,16]. However, for the best of our knowledge, the particular problem of the magnetophoresis in the polydisperse system remains under-investigated. Here, we will try to consider this problem theoretically.

## 2. Model and Methods

### 2.1. Model

We will consider magnetophoresis in a cylindrical layer filled with a ferrofluid. The source of the inhomogeneous field in this system is a current carrying wire, which is going through the layer symmetry axis. This configuration is particularly suited for the theoretical studies. First of all, due to the system symmetry, one can expect that local particle concentration will only depend on the radial coordinate, making the problem one-dimensional. Furthermore, most importantly, as the current field is azimuthal, the FF magnetization is always tangential to the layer borders—it means that the so-called demagnetization fields in the system are absent. Actually, ferrofluid mass transfer for such configuration has already been studied in [17,18]. However, the focus of these works was on the initial stages of the transfer process and not on the steady distribution. Besides, previously the problem was considered only in the limiting case of a linearly magnetized monodisperse FF in a weak field. Here, we will not put any restriction on the field value.

**Figure 1:**
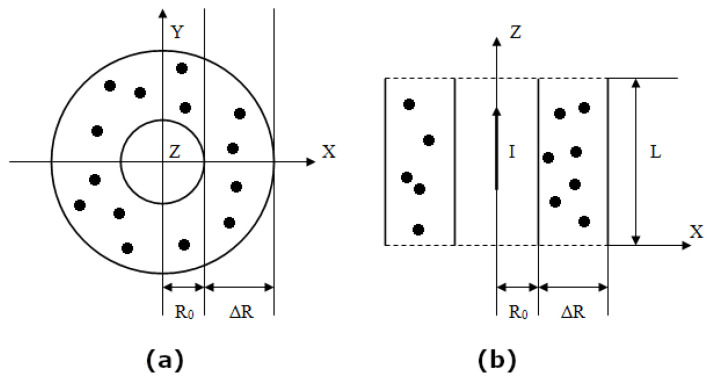
Schematic representation of the problem. (**a**) Transverse section. (**b**) Longitudinal section.

The system under study is a cylindrical layer filled with a suspension of MNPs in a nonmagnetic carrier fluid (see Figure 1). The layer is sandwiched between two coaxial cylinders impermeable to particles. The radius of the inner cylinder is $r=R_{0}$, and the radius of the external one is $r=R_{0}+\Delta R$. $r=\sqrt{X^{2}+Y^{2}}$ is the radial coordinate, let us introduce its dimensionless form

(1) $$\rho =\frac{r-R_{0}}{\Delta R},$$

ρ=0 corresponds to the inner wall of the cylindrical layer and ρ=1 corresponds to the outer wall. A constant current *I* passes through the axis of symmetry of the cylindrical layer (“*Z*-axis”). The current produces an azimuthal magnetic field

(2) $$\vec{H}=\frac{I}{2\pi r}{\hat{\vec{e}}}_{\varphi },$$

where ${\hat{\vec{e}}}_{\varphi }$ is the azimuthal unit vector. The system is thermostated and maintained at a constant temperature *T*. The particles are modeled as dipolar spheres with the diameter d=x+2σ, where *x* is the diameter of a uniformly magnetized metallic core and σ≃2nm is the combined thickness of the protective surfactant shell and the nonmagnetic layer on the particle surface. The particle magnetic moment is $m=M_{s}\pi x^{3}/6$, where $M_{s}$ is the saturation magnetization of the core material. The interaction between MNPs and the field can be described using the dimensionless Langevin parameter

(3) $$\begin{array}{c} \xi =\frac{\mu_{0}mH}{k_{B}T}=\frac{\xi_{0}}{\delta \rho +1}, \end{array}$$

(4) $$\begin{array}{c} \xi_{0}=\frac{\mu_{0}mI}{2\pi k_{B}TR_{0}}, \end{array}$$

(5) $$\begin{array}{c} \delta =\frac{\Delta R}{R_{0}}, \end{array}$$

where $\xi_{0}$ is the Langevin parameter near the inner wall of the cylindrical layer (it can be directly changed by changing the current amperage *I*), δ is the relative width of the layer, $\mu_{0}=1.26\times 10^{-6}$ H/m is the magnetic constant and $k_{B}=1.38\times 10^{-23}$ J/K is the Boltzmann constant. For T=300K, I=10A, $R_{0}=0.1\;\mathrm{mm}$ and x=10nm; the Langevin parameter is $\xi_{0}∼1$ for magnetite particles.

In general, MNPs can have different sizes and the size distribution can be described by some function f(x). The average concentration of particles in the system is $\bar{C}=N/V$ and the average volume fraction is $\bar{\Phi }={\langle v\rangle }_{x}\bar{C}$, where *N* is the total number of particles, *V* is the volume of the considered cylindrical layer, $v=\pi d^{3}/6$ is the particle volume, and ${\langle \ldots \rangle }_{x}=\int_{0}^{\infty }\ldots f\left(x\right)dx$ denotes the averaging over the size distribution.

At the initial moment of time, MNPs are uniformly distributed in the system, and there are no hydrodynamic flows. The magnetic field increases in the radial direction from the outer wall of the layer to the inner one. As a result, the particles will also begin to drift towards the wall until the magnetic flux at each point of the system is compensated by the diffusion flux directed against the local concentration gradient. After that, some equilibrium inhomogeneous radial concentration distribution C=C(ρ) will be achieved in the system, and the macroscopic transfer processes will eventually stop [19]. One can also expect that the particle size distribution f(x,ρ) will vary along the radial coordinate. The purpose of this work is to determine these equilibrium distributions for given values of $R_{0}$, ΔR and *I*. The problem will be solved using following assumptions:

- the particle volume fraction is small enough that the effect of interparticle interactions on the equilibrium particle distribution can be neglected, i.e., $\bar{\Phi },\Phi \left(\rho \right)\ll 1$, where Φ(ρ) is the local particle volume fraction (see additional discussion on the role of interparticle interactions in Section 3.3);
- the effect of the gravitational sedimentation on the system is small compared to the effect of magnetophoresis.

### 2.2. Continuous Mass Transfer Theory for the Monodisperse System

Here, we will employ the standard continuous medium approach that is often used for the description of mass transfer phenomena in FF [12,13,14,20,21]. Let us first consider the monodisperse system (i.e., all MNPs have identical magnetic core diameter *x*). The mass transfer equation for MNPs can be written simply as

(6) $$\frac{\partial C}{\partial t}=-\mathrm{div}\vec{j},$$

where $\vec{j}$ is the particle flux density. One can find in the literature rather complex expressions for $\vec{j}$, which take into account various interparticle interaction effects [19,22]. However, the assumption of noninteracting particles allows us to use the following simplified form:

(7) $$\begin{array}{c} \vec{j}={\vec{j}}_{D}+{\vec{j}}_{MP}, \end{array}$$

(8) $$\begin{array}{c} {\vec{j}}_{D}=-D\nabla C, \end{array}$$

(9) $$\begin{array}{c} {\vec{j}}_{MP}=b\mu_{0}mCL\left(\xi \right)\nabla H, \end{array}$$

where ${\vec{j}}_{D}$ is the diffusion flux density, *D* is the particle diffusion coefficient, *b* is the particle mobility, ${\vec{j}}_{MP}$ is the magnetophoretic flux density, L(ξ)=cothξ−1/ξ is the Langevin function, *H* is the macroscopic magnetic field in the system and in the general case it is the sum of the applied field and the demagnetization field. However, as was already mentioned, the configuration of our system excludes the appearance of demagnetization fields. Thus, the field in Equation (9) is the electric current field [Equation (2)]. We are only interested in the stationary solution of the mass transfer equation, which corresponds to the equilibrium distribution of MNPs. In this case, the particle flux density should be zero everywhere in the system volume, i.e., $\vec{j}=0$. Using this condition, Equation (7), the system symmetry (the field and the particle concentration can only vary along the radial coordinate) and the Einstein’s formula $D=bk_{B}T$, one arrives to the equation for the concentration profile

(10) $$\frac{1}{C}\frac{dC}{d\rho }=L\left(\xi \right)\frac{d\xi }{d\rho }.$$

The general solution of this equation can be written as

(11) $$\tilde{c}\left(\rho \right)=\frac{C(\rho )}{\bar{C}}=A\frac{\sinh\xi (\rho )}{\xi (\rho )},$$

where $\tilde{c}$ is the reduced particle concentration and *A* is the integration constant determined by the normalization condition

(12) $$\frac{\int_{V}\tilde{c}\left(\rho \right)dV}{V}=\frac{2\int_{R_{0}}^{R_{1}}\tilde{c}\left(\rho \right)rdr}{R_{1}^{2}-R_{0}^{2}}=\frac{\int_{0}^{1}\tilde{c}\left(\rho \right)\left(1+\rho \delta \right)d\rho }{1+\delta /2}=1,$$

where $R_{1}=R_{0}+\Delta R$. Combining Equations (11) and (12), one obtains for *A*

(13) $$\begin{array}{c} A=A\left(\xi_{0},\delta \right)=\frac{\delta (1+\delta /2)}{\xi_{0}^{2}}\frac{1}{B\left(\xi_{0}\right)-B\left(\xi_{0}/\left(1+\delta \right)\right)}, \end{array}$$

(14) $$\begin{array}{c} B\left(y\right)=\frac{y^{3}\mathrm{Chi}\left(y\right)-\left(y^{2}+2\right)\sinh y-y\cosh y}{6y^{3}}, \end{array}$$

(15) $$\begin{array}{c} \mathrm{Chi}\left(y\right)=E+\ln y+\int_{0}^{y}\frac{\cosh t-1}{t}dt, \end{array}$$

where Chi(y) is the hyperbolic cosine integral and E≈0.5772 is the Euler–Mascheroni constant.

### 2.3. Equilibrium Distributions of the Polydisperse System

Mass transfer equations for the polydisperse colloids in general are rather complex [23,24]. However, here we are only interested in the equilibrium distributions. We will employ an intuitive approach previously used in [25] to describe the concentration profile of a dilute polydisperse ferrofluid with weakly interacting MNPs. The assumption is that distributions of different particle fractions can be independently described using corresponding monodisperse solutions, and that the overall concentration profile C=C(ρ) is a simple superposition of these solutions:

(16) $$C\left(\rho \right)=\int_{x=0}^{x=\infty }dC\left(x,\rho \right)=\int_{x=0}^{x=\infty }\tilde{c}\left(x,\rho \right)d\bar{C}\left(x\right)=\bar{C}\int_{0}^{\infty }\tilde{c}\left(x,\rho \right)f\left(x\right)dx,$$

where dC(x,ρ) is the concentration profile of MNPs with the magnetic core diameter *x*, $d\bar{C}\left(x\right)=\bar{C}f\left(x\right)dx$ is the net concentration of these MNPs and $\tilde{c}\left(x,\rho \right)$ is the reduced concentration profile of these MNPs given by Equation (11) ($\tilde{c}$ in this equation is dependent on *x* via the parameter $\xi_{0}\propto m\propto x^{3}$).

Moving on, we can also write down the equilibrium particle size distribution f(x,ρ) at a given radius ρ. Mathematically this distribution can be defined as f(x,ρ)dx=dC(x,ρ)/C(ρ). Therefore,

(17) $$f\left(x,\rho \right)=\frac{\tilde{c}\left(x,\rho \right)f\left(x\right)}{\int_{0}^{\infty }\tilde{c}\left(x,\rho \right)f\left(x\right)dx}.$$

### 2.4. Langevin Dynamics

To ensure the accuracy of the obtained continuous theory results, we have used the Langevin dynamics simulations to study the equilibrium distributions of MNPs in a nonuniform field. The description of the simulated system mostly coincides with the one given in Section 2.1. Additionally, one-dimensional periodic boundary conditions are imposed along the *Z*-axis. The movement of the *i*-th particle is governed by a pair of Langevin equations:

(18) $$\begin{array}{c} {\dot{\vec{v}}}_{i}^{*}={\vec{F}}_{m,i}^{*}+{\vec{F}}_{iw,i}^{*}+{\vec{F}}_{ow,i}^{*}-\gamma^{*T}{\vec{v}}_{i}^{*}+{\vec{\eta }}_{i}^{*T}, \end{array}$$

(19) $$\begin{array}{c} J^{*}{\dot{\vec{\omega }}}_{i}^{*}={\hat{\vec{m}}}_{i}\times \vec{\xi }\left(\rho_{i}\right)-\gamma^{*R}{\vec{\omega }}_{i}^{*}+{\vec{\eta }}_{i}^{*R}, \end{array}$$

where asterisk denotes reduced quantities, *d* is used as a unit of length, particle mass M—as a unit of mass and the thermal energy $k_{B}T$—as a unit of energy. Thus, ${\vec{v}}_{i}^{*}={\vec{v}}_{i}\sqrt{M/k_{B}T}$ and ${\vec{\omega }}_{i}^{*}={\vec{\omega }}_{i}\sqrt{Md^{2}/k_{B}T}$ are the reduced linear and angular velocities, correspondingly; ${\vec{F}}_{m,i}^{*}={\vec{F}}_{m,i}d/k_{B}T=\left(\hat{\vec{m}}\cdot \nabla^{*}\right)\vec{\xi }\left(\rho_{i}\right)$ is the reduced magnetophoretic force; ${\vec{F}}_{iw,i}^{*}$ and ${\vec{F}}_{ow,i}^{*}$ are the reduced repulsion forces acting on the *i*-th particle from the inner and outer wall of the layers, correspondingly (to model this repulsion we have used the standard Weeks–Chandler–Anderson short-range potential [26]); ${\hat{\vec{m}}}_{i}={\vec{m}}_{i}/m$ is the unit vector along the particle magnetic moment; $J^{*}=J/Md^{2}$ is the reduced moment of inertia; $\gamma^{*T}=\gamma^{T}\sqrt{d^{2}/Mk_{B}T}$ and $\gamma^{*R}=\gamma^{R}\sqrt{1/d^{2}Mk_{B}T}$ are the reduced translational and rotational friction coefficient; and ${\vec{\eta }}_{i}^{*T}$ and ${\vec{\eta }}_{i}^{*R}$ are the random Gaussian force and torque, respectively, which have zero mean values and satisfy the standard fluctuation–dissipation relationship

(20) $$\begin{array}{c} \langle \eta_{i\alpha }^{*T(R)}\left(t_{1}^{*}\right)\eta_{j\beta }^{*T(R)}\left(t_{2}^{*}\right)\rangle =2\gamma^{*T(R)}\delta_{\alpha \beta }\delta_{ij}\delta^{*}\left(t_{1}^{*}-t_{2}^{*}\right), \end{array}$$

where 〈…〉 denote the thermodynamic ensemble average, the reduced time is $t^{*}=t\sqrt{k_{B}T/Md^{2}}$.

A home-written C++ realization of the Grønbech-Jensen and Farago leapfrog algorithm [27] is used for the numerical integration of Langevin equations. The input parameters of the simulation are $\xi_{0}$ and δ. Other parameters are typically fixed: $J^{*}=0.1$, $\gamma^{*R}=1$, $\gamma^{*T}=1$, N=1000, $\bar{\Phi }=0.001$. The dimensionless time step is $\Delta t^{*}=0.002$. Initially, all particles are distributed uniformally within the system. The equilibration period typically takes $10^{6}$ time steps, then another $10^{6}$ time steps are used to collect the data. To estimate concentration profiles, we have divided the system volume into multiple radial sublayers of equal width and calculated the time-averaged particle concentration within each sublayer.

## 3. Results and Discussion

### 3.1. Monodisperse System

**Figure 2:**
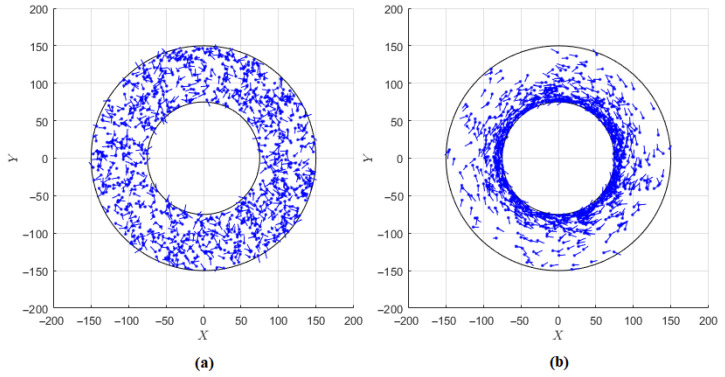
Snapshots of the equilibrated system obtained from the Langevin dynamics simulations for the relative layer width δ=1. Each blue point represents an instantaneous position of one of N=1000 particles on the XY-plane. Lines originating from points demonstrate the orientation of corresponding magnetic moments. (**a**) Electric current is absent ($\xi_{0}=0$). (**b**) Strong electric current is flowing along the Z-axis ($\xi_{0}=10$).

Figure 2 demonstrates some simulation snapshots of the equilibrated particle system both with and without the electrical current. The pictures match the expectations. In the absence of the current ($\xi_{0}=0$), particles are uniformly distributed within the cylindrical layer, the orientations of magnetic moments are random. However, at $\xi_{0}=10$ particles concentrate near the inner wall of the layer, and magnetic moments are predominantly oriented anti-clockwise, along the field lines.

**Figure 3:**
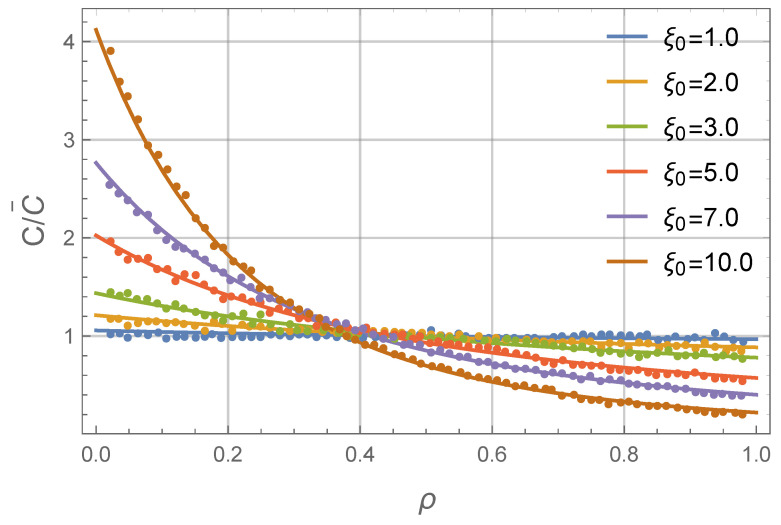
Concentration profiles of a monodisperse ferrofluid: reduced particle concentration $C/\bar{C}$ as a function of the dimensionless radius ρ. ρ=0 corresponds to the inner layer wall, ρ=1 corresponds to the outer layer wall. The relative layer width is δ=0.5. Solid lines are from Equation (11) and symbols are from the Langevin dynamics simulations. Different colors correspond to different values of the dimensionless amperage parameter $\xi_{0}$ (see Legend).

Figure 3 shows equilibrium concentration profiles for different values of $\xi_{0}$. First of all, an excellent agreement between theory and simulation can be seen. It verifies our theoretical calculations and also proves that the macroscopic continuous theory can successfully describe mass transfer in FF even at the microscopic scale (as actual linear sizes of the system in the Langevin dynamics simulations are of the order of $10^{2}x∼10^{3}\;\mathrm{nm}$). One can see from the profiles that an order of magnitude amperage increase can cause a substantial redistribution of MNPs in the system.

**Figure 4:**
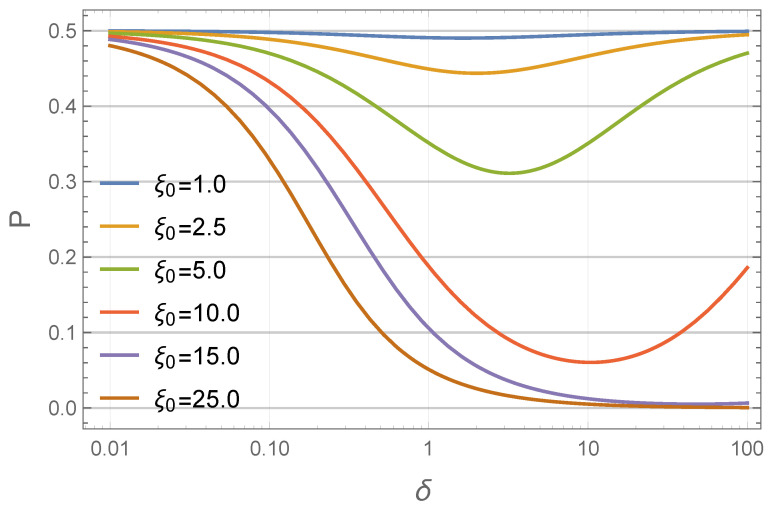
Effective height of the concentration profile *P* as a function of the relative layer width δ (as predicted by Equations (11) and (21)). Different colors correspond to different values of the dimensionless amperage parameter $\xi_{0}$ (see legend).

In order to investigate how the profile shape depends on $\xi_{0}$ and δ in more detail, let us introduce a dimensionless parameter

(21) $$P=\frac{\int_{0}^{1}\rho \tilde{c}\left(\rho \right)d\rho }{\int_{0}^{1}\tilde{c}\left(\rho \right)d\rho },$$

which can be considered as an “effective height” of the profile. For a uniform concentration distribution ($\tilde{c}\left(\rho \right)=1$), P=1/2. P<1/2 indicates that particles are concentrated near the inner wall. Smaller values of this parameter correspond to a greater degree of particle redistribution. Figure 4 shows *P* dependencies on δ for different values of $\xi_{0}$. One simple and obvious conclusion from these plots is that for a given system geometry (δ=const), a larger amperage causes a stronger particle inhomogeneity. For a given $\xi_{0}$, the dependence P=P(δ) is surprisingly non-monotonic.

**Figure 5:**
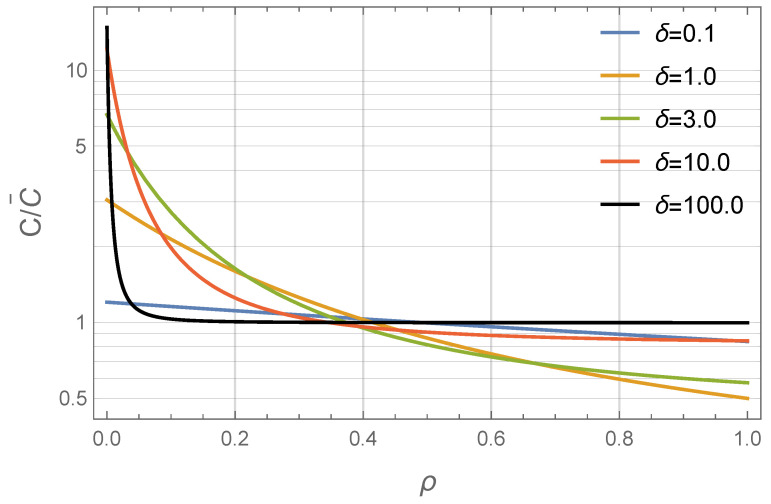
Concentration profiles of a monodisperse ferrofluid: reduced particle concentration $C/\bar{C}$ as a function of the dimensionless radius ρ (as predicted by Equation (11)). ρ=0 corresponds to the inner layer wall, ρ=1 corresponds to the outer layer wall. The dimensionless amperage parameter is $\xi_{0}=5$. Different colors correspond to different values of the relative layer width δ (see Legend).

To understand the P=P(δ) dependence, let us first consider Figure 5, which illustrates how the profile changes with changing the layer width δ. For small values of δ, particle concentration decreases gradually along the layer. However, as δ increases, the profile shape changes qualitatively—now the particle concentration is highly non-monotonic in the vicinity of the inner wall, but remains nearly constant ($C≲\bar{C}$) elsewhere. This is due to the nonlinear coordinate dependence of the magnetophoretic force. Indeed, the equilibrium force on the particle is $F_{m}=\mu_{0}mL\left(\xi \right)\nabla H\propto L\left(\xi_{0}/\left(1+\delta \rho \right)\right)/{\left(1+\delta \rho \right)}^{2}$. Thus, for δ≪1, the force practically does not change along the layer—this situation qualitatively resembles the effect of the gravitational field on the horizontal flat layer. The particle distribution in this case resembles the barometric one. For δ≫1, the force has its maximum value at ρ=0, but can become almost negligible at ρ≲1, resulting in the weak particle drift within the overall system volume.

### 3.2. Polydisperse System

Now, let us move on to the the more complex case of a polydisperse system. The profile function given by Equation (11) depends on the magnetic core diameter through the amperage parameter $\xi_{0}\propto x^{3}$. Thus, even for particle fractions with close sizes (say, x=10 nm and x=15 nm) the fraction concentration profiles can be quite different.

To be specific, let us consider the gamma-distribution of magnetic cores, which is often used to describe properties of industrial FFs [28]. The distribution function can be written as

(22) $$f\left(x\right)=\frac{x^{\alpha }\exp\left(-x/x_{0}\right)}{x_{0}^{\alpha +1}\Gamma \left(\alpha +1\right)},$$

where Γ(x) is the gamma function, $x_{0}$ and α are the distribution parameters, which are directly connected to the average diameter and the relative distribution width as

(23) $$\begin{array}{c} {\langle x\rangle }_{x}=x_{0}\left(\alpha +1\right), \end{array}$$

(24) $$\begin{array}{c} \Delta_{x}=\sqrt{\frac{{\langle x^{2}\rangle }_{x}}{{\langle x\rangle }_{x}^{2}}-1}=\frac{1}{\sqrt{\alpha +1}}. \end{array}$$

**Figure 6:**
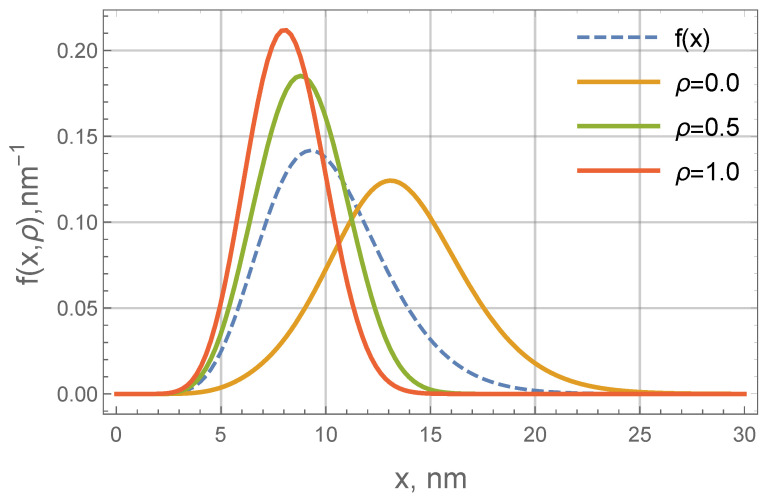
Particle size distributions of a polydisperse ferrofluid, placed inside the cylindrical layer with the inner radius $R_{0}=0.1\;\mathrm{mm}$ and the relative width δ=1. Dashed line is the original distribution of a uniform ferrofluid (i.e., the distribution in the absence of electric current, at I=0)—this is a gamma-distribution (Equation (22)) with parameters $x_{0}=0.84$ nm and α=11.06. Solid lines are equilibrium size distributions at different distances from the current carrying wire at I=50 A and T=300 K (see Legend). They are calculated using Equations (11) and (17). ρ=0 corresponds to the inner wall, ρ=1 corresponds to the outer wall.

**Figure 7:**
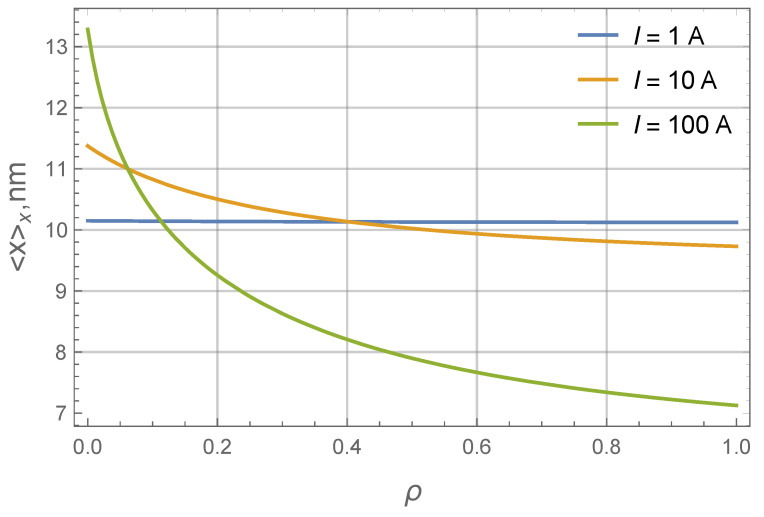
Average diameter of the particle magnetic core ${\langle x\rangle }_{x}$ as a function of the dimensionless radius ρ at $R_{0}=0.1\;\mathrm{mm}$, T=300 K and δ=1. Different colors correspond to different amperage values (see Legend). The size distribution at I=0 is modeled by the gamma-distribution (22) with the average diameter ${\langle x\rangle }_{x}=10.1$ nm. Values of ${\langle x\rangle }_{x}$ at different ρ are calculated numerically from distributions (17) using Equation (11) to describe profiles of individual fractions.

As an example, we will use values $x_{0}=0.84$ nm and α=11.06. They correspond to ${\langle x\rangle }_{x}=10.1$ nm and $\Delta_{x}=0.29$. Magnetite-based FF with a similar size distribution was experimentally studied in [29], its properties are close to many typical industrial FFs. Further on, let us use δ=1, $R_{0}=0.1\;\mathrm{mm}$ (this is close to the geometry used experimentally in [18]) and T=300 K. As was estimated previously, for these parameters, $\xi_{0}\left(x=10\;\mathrm{nm}\right)\approx 1$ at I=10 A. Figure 6 shows how the size distribution is changing along the layer at I=50 A. The size distributions at different points clearly differ from the original gamma distribution. The largest particles strongly tend to concentrate near the inner wall. As a result, the average diameter decreases as ρ goes from one to zero. In more detail, the nonlinear coordinate dependency of the average diameter is shown in Figure 7.

### 3.3. On the Role of Interparticle Interactions

As was mentioned previously, in this work we were focused on the low-concentration systems, and thus interparticle interactions were ignored. However, as we also considered polydisperse ferrofluids containing a certain amount of comparatively large particles, some additional clarification is required.

For a proper theoretical handling of interparticle interactions in ferrofluids one needs to consider at least two independent dimensionless parameters—the particle volume fraction Φ and the so-called dipolar coupling constant λ [22]. The latter can be defined as

(25) $$\lambda =\frac{\mu_{0}}{4\pi }\frac{m^{2}}{d^{3}k_{B}T}.$$

The coupling constant is the ratio between the dipole-dipole interaction energy of two adjacent particles and the thermal energy. Sometimes it is called the aggregation parameter, since at high values of λ magnetic particles tend to form various types of aggregates, including chains, rings and branching structures [30,31]. At high λ aggregation takes place in a broad concentration range, including extremely low values of Φ [32]. In turn, the effect of aggregates on the mass-transfer properties of ferrofluids is known to be significant and mathematically cumbersome to account [33]. Luckily, high dipolar coupling constants are not typical for industrial ferrofluids [28]. Let us make some estimations. MNPs in a dilute ferrofluid are able to form chains starting from $\lambda^{*}≃4$ [16,30,31]. For magnetite nanoparticles ($M_{s}=450$ kA/m) with the protective surfactant shell of thickness σ=2 nm at temperature T=300 K, this critical coupling constant corresponds to the magnetic core diameter $x^{*}≃18$ nm. For a gamma-distribution considered in a previous section ($x_{0}=0.84$ nm, α=11.06), the fraction of particles having large enough magnetic cores is $\int_{x^{*}}^{\infty }f\left(x\right)dx≃0.01$. It means that only about one percent of particles in this system are capable of forming chains. The average coupling constant for the whole polydisperse system can be estimated as $\lambda =\left(\mu_{0}/4\pi k_{B}T\right){\langle m{\left(x\right)}^{2}\rangle }_{x}/{\langle {\left(x+2\sigma \right)}^{3}\rangle }_{x}≃1.35$. Thus, we believe, that the neglect of aggregation processes in this case is well justified.

In a non-aggregated ferrofluid, dipole–dipole interactions can be accurately taken into account within mean-field-like approaches. Often in this case overall interaction effects are described using just a single dimensionless parameter—the so-called Langevin susceptibility $\chi_{L}=8\lambda \Phi$ [19]. Using λ=1.35 and $\bar{\Phi }=0.001$ (the value we used in simulations), we get for our system $\chi_{L}∼10^{-2}$. This is a small enough value to ignore interactions completely. Obviously, for systems with the particle volume fraction reaching several percent or more, interactions can actually influence the concentration profile shape. Based on previous works, in which the sedimentation of interacting MNPs was modeled, we can expect that the interactions will lead to a larger concentration inhomogeneity and to a stronger degree of particle separation within the investigated cylindrical layer [16,22]. However, this problem is outside the scope of the present paper and is left for further studies.

## 4. Conclusions

In this work, the equilibrium spacial distribution of MNPs near the linear current carrying wire was investigated theoretically. An exact analytical solution for the profile shape was obtained within the continuous theory of a mass transfer in a dilute monodisperse ferrofluid. The applicability of this solution was tested using Langevin dynamics simulations. It was shown, that for a given current amperage the equilibrium profile changes qualitatively depending on the system geometry. If the cylindrical ferrofluid-filled gap around the wire is narrow enough, the magnetophoretic force acting on particles is nearly constant within the system volume and the radial particle distribution resembles the barometric one. However, if the gap is wide, the non-monotonic distribution mostly exist only in the direct vicinity of the wire.

It is shown that the profile shape depends strongly on the particle diameter. As a result, the concentration distribution of a polydisperse ferrofluid becomes especially complex. More than that, the particle size distribution at different points of the system is different and generally does not coincide with the initial size distribution of a uniform ferrofluid: near the wire the average particle diameter becomes larger. Potentially, this feature can be used to manufacture ferrofluids with a finely tuned particle size distribution.

## Data Availability

The data presented in this study are available on request from the corresponding author.

## Abbreviations

The following abbreviations are used in this manuscript:

FF	ferrofluid
MNP	magnetic nanoparticle
